# Dynamic changes in circulating microRNAs during oral glucose tolerance testing support their potential as diagnostic and monitoring biomarkers in cystic fibrosis-related diabetes

**DOI:** 10.1007/s00125-025-06645-7

**Published:** 2026-01-07

**Authors:** Efraim Westholm, Alexandros Karagiannopoulos, Bibi U. Nielsen, James A. M. Shaw, Anna Wendt, Daniel Faurholt-Jepsen, Lena Eliasson

**Affiliations:** 1 Islet Cell Exocytosis, Lund University Diabetes Centre (LUDC), Department of Clinical Sciences-Malmö, Lund University, Malmö, Sweden; 2 Clinical Research Centre (CRC), Skåne University Hospital, Malmö, Sweden; 3 Cystic Fibrosis Centre Copenhagen, Department of Infectious Diseases, Copenhagen University Hospital - Rigshospitalet, Copenhagen, Denmark; 4 Translational and Clinical Research Institute, Newcastle University, Newcastle upon Tyne, UK; 5 Department of Clinical Medicine, University of Copenhagen, Copenhagen, Denmark

**Keywords:** Beta cell, CFRD, Insulin secretion, Liver marker, miRNA, Oral glucose tolerance test

## Abstract

**Aims/hypothesis:**

MicroRNAs are potential predictors and mediators of metabolic disease. Cystic fibrosis-related diabetes (CFRD) is the most common comorbidity in cystic fibrosis (CF). Here we aimed to investigate serum microRNAs in individuals with CF and differing glucose tolerance status. Specifically, we hypothesised that the circulating microRNA profile varies depending on glucose tolerance status and can change rapidly in response to a glucose challenge in individuals with CF.

**Methods:**

We studied a cohort of 93 adult Danish participants with CF from four glucose tolerance categories: normal glucose tolerance, indeterminate glucose tolerance, impaired glucose tolerance and CFRD. In a cross-sectional design we sampled during an OGTT at baseline, 10 min, 30 min, 60 min and 180 min. A total serum microRNA sequencing was performed using baseline and 60 min samples from 12 selected individuals, three from each category. We identified 16 candidate microRNAs, and these were further investigated in the full cohort at all OGTT timepoints using a locked nucleic acid reverse transcription quantitative PCR assay. Three microRNAs were selected for in-depth assessment including impact on insulin secretion.

**Results:**

We identified four microRNAs differentially expressed at baseline (miR-34a-5p, miR-122-5p, miR-885-3 and miR-885-5p) and 12 with differential expression changes in response to glucose ingestion. Locked nucleic acid reverse transcription quantitative PCR assay validated the results of eight of these microRNAs and miR-34a-5p, miR-122-5p and miR-223-3p were selected for in-depth assessment. MiR-34a-5p and miR-122-5p were elevated at baseline in indeterminate glucose tolerance and CFRD and were associated with elevated liver damage markers. MiR-223-3p was differentially expressed during the OGTT, with different patterns depending on glucose tolerance state. Glucose-stimulated insulin secretion was increased after overexpression of miR-122-5p or miR-223-3p and cell viability was decreased after overexpression of miR-34a-5p in insulin-secreting cells.

**Conclusions/interpretation:**

MiR-34a-5p and miR-122-5p show potential as biomarkers for CFRD development and liver damage. MiR-122-5p and miR-223-3p could mitigate CFRD development by increasing the secretory capacity of the beta cells while miR-34a-5p might propagate CFRD development by reducing cell viability. We propose that circulating microRNAs can serve as biomarkers for CF complications. We further advocate that circulating microRNAs can play a part in the intricate crosstalk between metabolic organs and the endocrine pancreas in health and disease.

**Graphical Abstract:**

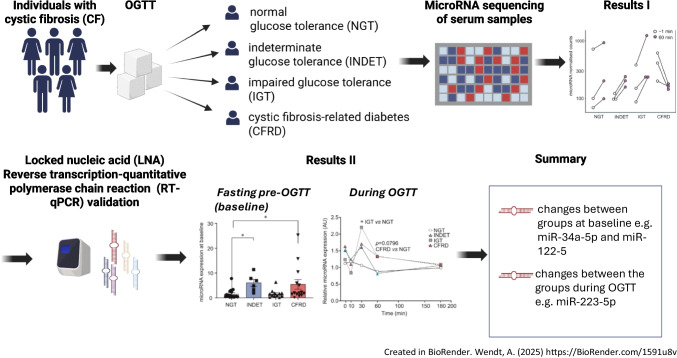

**Supplementary Information:**

The online version contains peer-reviewed but unedited supplementary material available at 10.1007/s00125-025-06645-7.


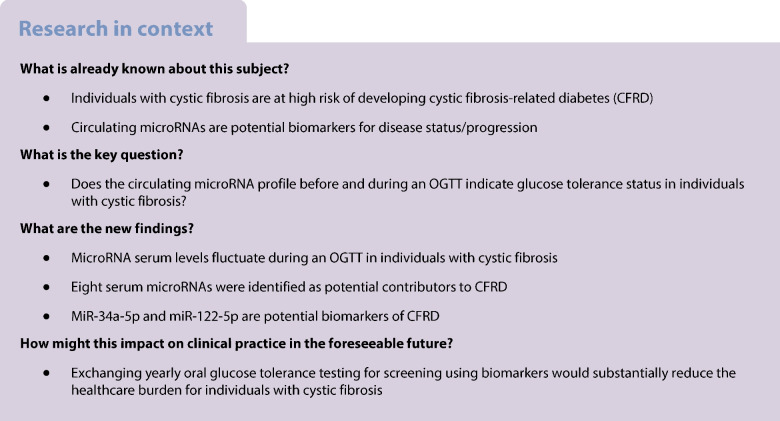


## Introduction

Cystic fibrosis-related diabetes (CFRD) is the most common comorbidity in individuals with cystic fibrosis (CF), affecting at least 50% of the adult CF population [1]. Acquiring CFRD is associated with a ~6-fold increase of morbidity and mortality [2]. The pathogenesis of CFRD is associated with impaired pancreatic islet function [3, 4] and reduced/absent first-phase insulin secretion [5]. The mechanisms behind the decline in islet function are debated but could be due to the progressive destruction of the pancreas observed in CF individuals and/or to the effects of *CFTR* (encoding cystic fibrosis transmembrane conductance regulator [CFTR]) loss-of-function variants in pancreatic islet cells themselves [6–8]. Additionally, hepatic insulin resistance and reduced peripheral insulin sensitivity can contribute to CFRD [9–11].

MicroRNAs are short, non-coding RNA molecules involved in the regulation of cellular processes [12]. It has been shown that microRNAs can circulate in the blood enclosed in exosomes or bound to proteins such as Argonaute proteins. Circulating microRNAs are important biomarkers for disease status/progression but are also suggested as mediators of intercellular and interorgan crosstalk [13–15]. For example, exosomal release of miR-99b from adipose tissue negatively regulates liver metabolism leading to impaired blood glucose management [16]. In type 1 diabetes, exosomes from T lymphocytes carrying miR-142-3p, miR-142-5p and miR-155 are taken up by beta cells, leading to apoptosis and reduced insulin content [17].

We hypothesise that the circulating microRNA profile differs with glucose tolerance status in individuals with CF, and that the circulating microRNA profile can change rapidly in response to environmental challenges such as increased blood glucose levels. To investigate these possibilities, we analysed circulating microRNAs in OGTT serum samples from CF individuals with incremental progression of glucose intolerance. We evaluated differential expression of total serum microRNA levels at baseline, as well as microRNA expression changes during the OGTT. With this approach we identified eight microRNAs of interest. Of these, three differentially regulated microRNAs were selected for deeper assessment.

## Methods

### Ethics statement

Ethical permission to conduct the study was given by Scientific Ethics Committees for the Capital Region of Denmark, Copenhagen, Denmark (H-19085530) and informed written consent was acquired from each participant. Ethical approval for the microRNA analysis and data handling was granted by Etikprövningsmyndigheten, Sweden (Dnr-2021-04670).

### Total serum microRNA sequencing

#### Participant selection and microRNA extraction

Samples were collected from 93 adult Danish participants with CF in a cross-sectional OGTT study using the inclusion criteria of age ≥18 years and a confirmed CF diagnosis. Race and ethnicity data are not included in this study. These data are not routinely collected in national healthcare registries in Denmark because of their sensitivity and in this study there is no scientific rational for their inclusion. Differences between male and female participants were not investigated due to the small sample size. Except for individuals with lung transplantation and pregnant women, all adult participants without CFRD and participants with CFRD, who had participated in prior research at the Copenhagen research centre, were invited to participate [18]. Sampling was performed at the timepoints: −1 min (baseline), 10 min, 30 min, 60 min and 180 min. The cohort was divided into four glucose tolerance categories: normal glucose tolerance (NGT; all blood glucose levels <11.1 mmol/l, 2 h glucose <7.8 mmol/l), indeterminate glucose tolerance (INDET; mid-OGTT glucose ≥11.1 mmol/l and 2 h glucose <7.8 mmol/l), impaired glucose tolerance (IGT; 2 h glucose 7.8–11.0 mmol/l) and CFRD (2 h glucose ≥11.1 mmol/l) [19]. Samples from 12 individuals were selected for microRNA sequencing of serum from the baseline and 60 min timepoints. The 12 individuals were selected to ensure equal distribution of glucose intolerance (three from each category), identical genotype (homozygous for the F508del variant) and similar modulator treatment status (no exposure to highly effective CFTR modulator therapy). Total serum microRNA (including exosomal microRNA) was extracted from 200 μl of serum using the Qiagen miRNeasy Serum/Plasma Kit (cat. no.: 217184, Hilden, Germany). Collection of participant characteristics and OGTT samples has previously been described [18]. Randomisation or masking of samples was not performed.

#### MicroRNA library preparation and sequencing

Qiagen’s QIAseq microRNA Library Kit (cat. no.: 331502, Hilden, Germany) was used to generate microRNA libraries and indexing was performed with QIAseq microRNA NGS 48 Index IL (cat. no.: 331595). Library size control and quality control (QC) were performed on a 2200 TapeStation System (Agilent, Santa Clara, USA) to ensure correct library size at ~180 bp and no formation of unspecific PCR products. Library concentrations were determined with a Qubit 2.0 (v3.11) fluorometer (Thermo Scientific, Waltham, USA) and a pooled library with 1.2 pmol/l concentration was sequenced using an Illumina NextSeq 500/550 High Output Kit v2.5, 75 Cycles (cat. no.: 20024906, Illumina, San Diego, USA). Libraries were sequenced on an Illumina platform, NextSeq 500 system (cat. no.: SY-415-1001, Illumina).

#### Small RNA sequencing

The quality of the sequencing reads was assessed with FastQC (v0.11) [20], before processing with an in-house bioinformatics pipeline. First, 3′ adapters were identified and removed while simultaneously storing the information of the 12 bp unique molecular identifiers (UMIs) introduced by the QIAseq microRNA Library Kit. This was done by using UMI-Tools (v1.0.1) [21]. Next, the 5′ adapters were removed with Cutadapt (v3.7) [22] with a maximum error rate = 0.2. Low-quality bases were further removed with Trimmomatic (v0.39) [23] by applying a sliding window filtering of 4 bp and a quality threshold below 25. Reads less than 18 bp and more than 30 kb were discarded. Reads were then mapped to the human miRNAome (miRbase v22) with the STAR aligner [24] allowing for two mismatches, and additional parameters included end-to-end alignment, maximum number of multiple alignments = 10 and minimum number of nucleotides to be aligned = 16. After excluding reads mapped to the reverse strand with featureCounts (Subread v1.6.4) [25], reads with identical UMIs were represented by a single read before all the reads were counted.

### MicroRNA LNA RT-qPCR study

#### Custom LNA RT-qPCR plate design

To validate and further investigate the microRNAs identified in the total serum microRNA sequencing study, a custom reverse transcription quantitative PCR (RT-qPCR) plate with pre-loaded locked nucleic acid (LNA) primers (electronic supplementary material [ESM] Table 1) was designed in Qiagen’s GeneGlobe web tool (URL: https://geneglobe.qiagen.com/us/product-groups/mircury-lna-mirna-custom-pcr-panels). MiR-23a-3p was added for haemolysis assessment and as a serum control. A set of short synthetic spike-in RNAs were added for extraction control (UniSp2, 4, 5), interplate calibration (UniSp3) and PCR control (UniSp6). See  ESM Table 1 for the full custom 384-well plate layout.

#### MicroRNA extraction, RT-qPCR protocol and sample QC

Total RNA was extracted from 200 μl of serum using the Qiagen miRNeasy Serum/Plasma Advanced Kit (cat. no.: 217204) with the addition of spike-in extraction controls (cat. no.: 339390). MicroRNA cDNA was generated using a general microRNA reverse transcription reaction with the miRCURY LNA RT Kit (cat. no.: 339340), using 4 μl of eluate. Real-time quantitative PCR was performed using the miRCURY SYBR Green PCR Kit (cat. no.: 339347). In total, 418 samples from 93 individuals were analysed across 28 plates. Plates were run on a Quantstudio Flex 7 (cat. no.: 4485701). Sample QC entailed exclusion of samples with reverse transcription PCR inhibition (UniSp6 C_t_ values >23), requirement of good extraction efficiency (no difference greater than ΔC_t_ of 3 between each extraction control, UniSp2, 4 or 5, across all OGTT samples in the same participant) and minimal haemolysis (ΔCt=Ct[miR-23a-3p] − Ct[miR-451a] <7). For inclusion of an individual, the baseline sample and two additional samples in the OGTT time series had to pass sample QC. The timepoint samples, three or more, included for each individual varied. Differential expression analysis was therefore performed for each timepoint separately.

### Bioinformatic microRNA target analysis

To identify potential mRNA targets of selected microRNAs, data on experimentally validated microRNA targets were derived from TarBase (v8) [26] and MiRTarbase (v8) databases [27]. To increase the confidence of potential interaction, only mRNA targets expressed in human islets were retained. For this, data from a study of 219 non-CF human islet donors were used [28]. Association of targets with type 2 diabetes was assessed using genes found to be differentially expressed in type 2 diabetes vs non-diabetic islets in multiple studies [28–35]. To find enriched pathways in which mRNA targets are involved, we performed over-representation analysis with WebGestatlR (v0.4.6) [36] using the non-redundant Gene Ontology (Biological Process) database.

### In vitro follow-up study: INS-1 832/13 cell culture, microRNA overexpression, glucose-stimulated insulin secretion and MTS assay

INS-1 832/13 cells were cultured in 48-well plates, transfected with pre-microRNAs and assayed for glucose-stimulated insulin secretion as described previously [37]. Cells were regularly tested for mycoplasma contamination. Concentrations used for overexpression were: 1 nmol/l Pre-miR-34a-5p (cat. no.: PM11030, Ambion), 0.01 nmol/l Pre-miR-122-5p (cat. no.: PM11012, Ambion) and 0.1 nmol/l Pre-miR-223-3p (cat. no.: PM12301, Ambion). Concentrations were chosen based on dose–response experiments and showed an overexpression of 1000–10,000 greater than control, and/or C_t_ values around 19–23. Secreted and total insulin were measured using Mercodia’s high-range rat insulin ELISA kit (cat. no.: 10-1145-01). Cell lysates were collected in RIPA buffer (150 nmol/l NaCl, 1% TritonX-100, 0.1% SDS, 50 mmol/l Tris-Cl, pH 8). Total protein was measured using Pierce BCA Protein Assay Kit (cat. no.: 23225, Waltham, MA, USA). Transfection efficiency was measured using RT-qPCR with TaqMan assays (miR-34a-5p: TM000426; miR-122-5p: TM002245; miR-223-3p: TM002295; Waltham, MA, USA) and normalised to small nuclear RNAs U6 (TM001973) and U87 (TM001712). Cell viability was measured using an MTS assay (cat. no.: G3582, Promega).

### Statistical analyses

#### Differential microRNA response analysis in the total serum microRNA sequencing study

Differential response analysis was performed using the normalised expression levels obtained using the DESeq2 tool for R (v1.34) [38]. Only expressed microRNAs, defined by more than five normalised counts in at least 50% of the samples, were included in the analyses. Changes in microRNA levels in response to glucose ingestion were compared between the NGT group and the three other groups using a generalised linear model, where interaction of condition (NGT/INDET/IGT/CFRD) and OGTT timepoint (−1/60 min) was tested with the Wald significance test as applied by DESeq2. The model was corrected for participant variability.

#### LNA RT-qPCR data processing on the GeneGlobe platform, microRNA analyses, Spearman correlations and cell line data

All included sample data were uploaded to the GeneGlobe platform (URL: https://geneglobe.qiagen.com/se/analyse) for adjustment to the UniSp3 interplate calibrator. MicroRNA expression for each group at each timepoint was normalised to the mean of endogenous serum microRNA controls miR-16-5p, miR-23a-3p and miR-486-5p for all samples at that group/timepoint, and differential expression was visualised with volcano plots [39, 40]. MiR-23a-3p was recommended by the manufacturer, whereas miR-16-5p and miR-486-5p were selected based on their high abundance and stability in the total serum microRNA sequencing (did not change between baseline and 60 min timepoints in the OGTT or between groups) [41]. It was ascertained that the levels of the three microRNAs were not related to age, sex or CFTR modulator treatment status, and we verified that the levels of the endogenous serum microRNA controls did not change throughout the OGTT in the LNA RT-qPCR measurements. Volcano plots for LNA RT-qPCR data were generated on the Qiagen GeneGlobe platform and analysed using Student’s *t* test according to instructions at the GeneGlobe platform. For analyses across all four groups, the geometric mean of the C_t_ values for serum endogenous controls was subtracted from the microRNA C_t_ value for in-sample normalisation, whereafter the geometric mean of the microRNA in the NGT baseline group was subtracted for between-group normalisation. MicroRNA expression as the ${2}^{-\Delta \Delta {\mathrm{C}}_{\mathrm{t}}}$ value at baseline and throughout the OGTT was plotted and analysed in GraphPad Prism v.9 using one-way ANOVA or mixed-effects analyses as indicated. MicroRNA ${2}^{-\Delta \Delta {\mathrm{C}}_{\mathrm{t}}}$ values were plotted against blood glucose levels, insulin levels, and liver damage and inflammation markers. Spearman correlations were used to assess associations between microRNA values and blood analytes.

Cell line data were analysed in GraphPad Prism v.9 using paired Student’s *t* test or two-way ANOVA as indicated.

## Results

### Identification of abundant microRNAs in serum from individuals with CF

We first conducted an unbiased total serum microRNA screen using small RNA sequencing on serum samples collected at baseline and 60 min during an OGTT from 12 individuals with CF carefully selected to be representative of those in the overall cohort in the different glucose tolerance categories (three with NGT, three with INDET, three with IGT and three with CFRD; see the Methods and Table 1). We reasoned that the benefits of this unbiased approach in combination with the novelty of investigating acute effects of a glucose challenge outweighed the disadvantage of the small number of individuals investigated.

**Table 1:** Clinical characteristics of the 12 individuals in the total serum microRNA sequencing

Characteristic	NGT	INDET	IGT	CFRD
*N* (male/female)	3 (1/2)	3 (3/0)	3 (1/2)	3 (1/2)
Age (years)	29.7	30.0	36.7	38.7
BMI (kg/m^2^)	22.0	23.7	20.8	22.0
Insulin (pmol/l)	26.7	45.7	27.2	23.7
Glucose (mmol/l)	4.7	5.3	5.3	8.3
CRP (mg/l)	11.3	3.0	9.0	2.3
LPK (10^6^ cells/l)	6.3	6.2	6.6	7.3

Data are shown as mean at baseline

**Fig. 1:**
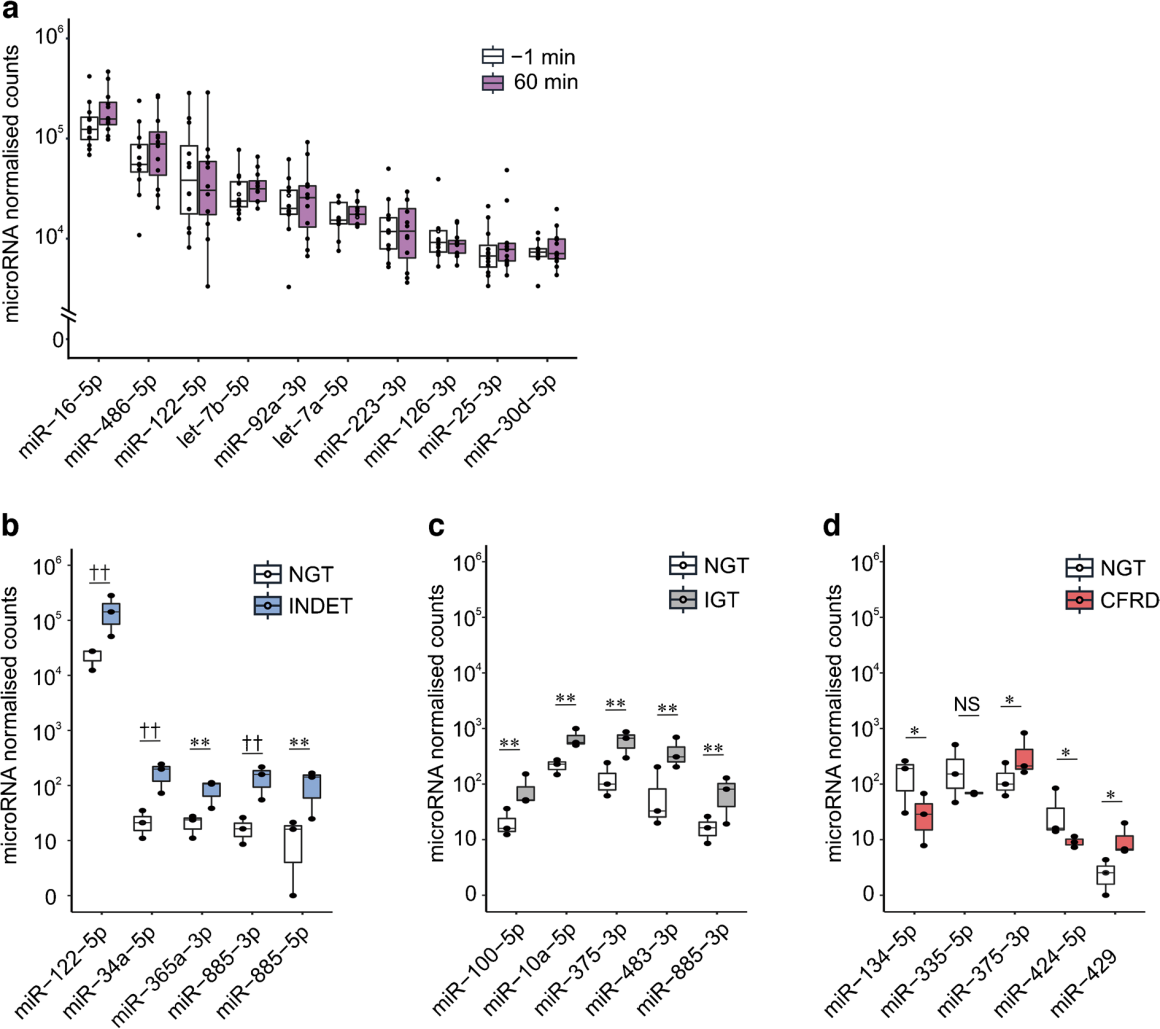
Abundance of microRNAs in serum measured by microRNA sequencing. (**a**) Box plot displaying the top ten most abundant microRNAs in serum microRNA sequencing by normalised DESeq2 counts. White boxes show all 12 samples at baseline (−1 min) from all four glucose tolerance categories (three NGT individuals, three INDET, three IGT, three CFRD). Purple boxes show all 12 samples at the 60 min timepoint. (**b**) Box plot showing the top five differentially expressed microRNAs at baseline in pairwise comparisons between NGT and INDET. (**c**) Same as (**b**), but for NGT and IGT. (**d**) Same as (**b**), but for NGT and CFRD. Boxplots represent the median with first and third quartiles (25th and 75th percentiles). Whiskers show minimum and maximum values within 1.5×IQR. Statistical significance was assessed with Wald’s test using DESeq2. ^††^FDR-adjusted *p*<0.01, **nominal *p*<0.01, *nominal *p*<0.05

We found 275 robustly expressed microRNAs and identified miR-16-5p, miR-486-5p and miR-122-5p as the three most abundant microRNAs in all samples (Fig. 1a). When we ranked the most differentially expressed microRNAs at baseline, miR-122-5p, together with miR-34a and miR-885-3p, showed significant (false discovery rate [FDR]-adjusted *p*<0.05) differences in the INDET vs the NGT group (Fig. 1b, ESM Table 2) and miR-885-5p showed nominal significant difference in both the INDET and the IGT groups vs the NGT group (Fig. 1c). We therefore chose these four microRNAs to be quantitatively assessed at all OGTT timepoints in the overall cohort.

### Differential regulation of 12 microRNAs by an oral glucose challenge

**Fig. 2:**
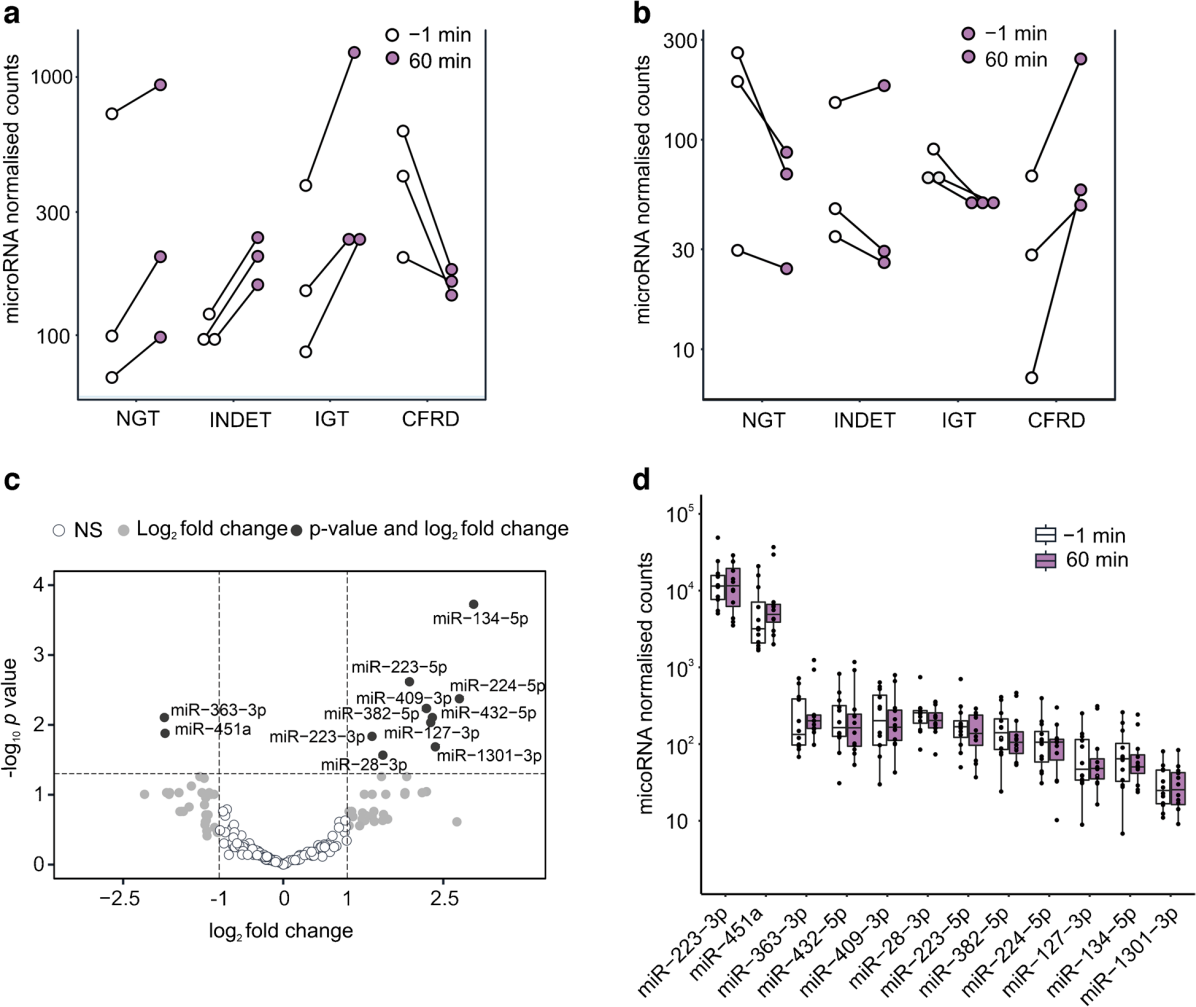
Differential response analysis identifies microRNA expression level changes after glucose intake. (**a**, **b**) Dot plots showing dynamic expression changes for miR-363-3p (**a**) and miR-134-5p (**b**). Connected dots show separate individuals within the four groups. White dots show the baseline (−1 min) and purple dots show the 60 min timepoint. (**c**) Volcano plot showing differential expression changes of microRNAs between NGT and CFRD following glucose intake. Top-left microRNAs have a negative trend in CFRD and a positive trend in NGT [see miR-363-3p in (**a**)] and top-right microRNAs have a positive trend in CFRD and a negative trend in NGT [see miR-134-5p in (**b**)]. Cut-offs at ±2-fold change and FDR-adjusted *p*<0.05. White dots: no fold change, NS; grey dots: fold change, NS; black dots: fold change and *p*<0.05. (**d**) Abundance plot for all 12 microRNAs with differential response identified in (**c**)

Next, we examined whether the 275 serum microRNAs from the total serum microRNA sequencing changed between baseline and 60 min in the OGTT. An example of the analysis is shown in Fig. 2a, b. Here the miR-363-3p levels changed positively between baseline and the 60 min timepoint in the NGT group whereas they changed negatively between the same timepoints in the CFRD group. For miR-134-5p, the levels changed in opposite directions. In a differential response analysis to glucose (comparing the change in microRNA expression between baseline and 60 min in NGT vs the three other groups; Fig. 2c, ESM Fig. 1), two microRNAs showed a negative response (miR-363-3p and miR-451a; Fig. 2a, ESM Fig. 1a) and ten microRNAs showed a positive response to glucose in CFRD (miR-28-3p, miR-127-3p, miR-134-5p, miR-223-3p, miR-223-5p, miR-224-5p, miR-382-5p, miR-409-3p, miR-432-5p and miR-1301-3p; Fig. 2b, ESM Fig. 1b). There were no microRNAs with a significant differential response in INDET or IGT (ESM Fig. 1c). All 12 microRNAs with differential expression in response to glucose during the OGTT in CFRD were selected for inclusion in the LNA RT-qPCR in the overall cohort (Fig. 2d).

### Analysis of differential expression of candidate microRNAs within the overall cohort at each OGTT timepoint

**Fig. 3:**
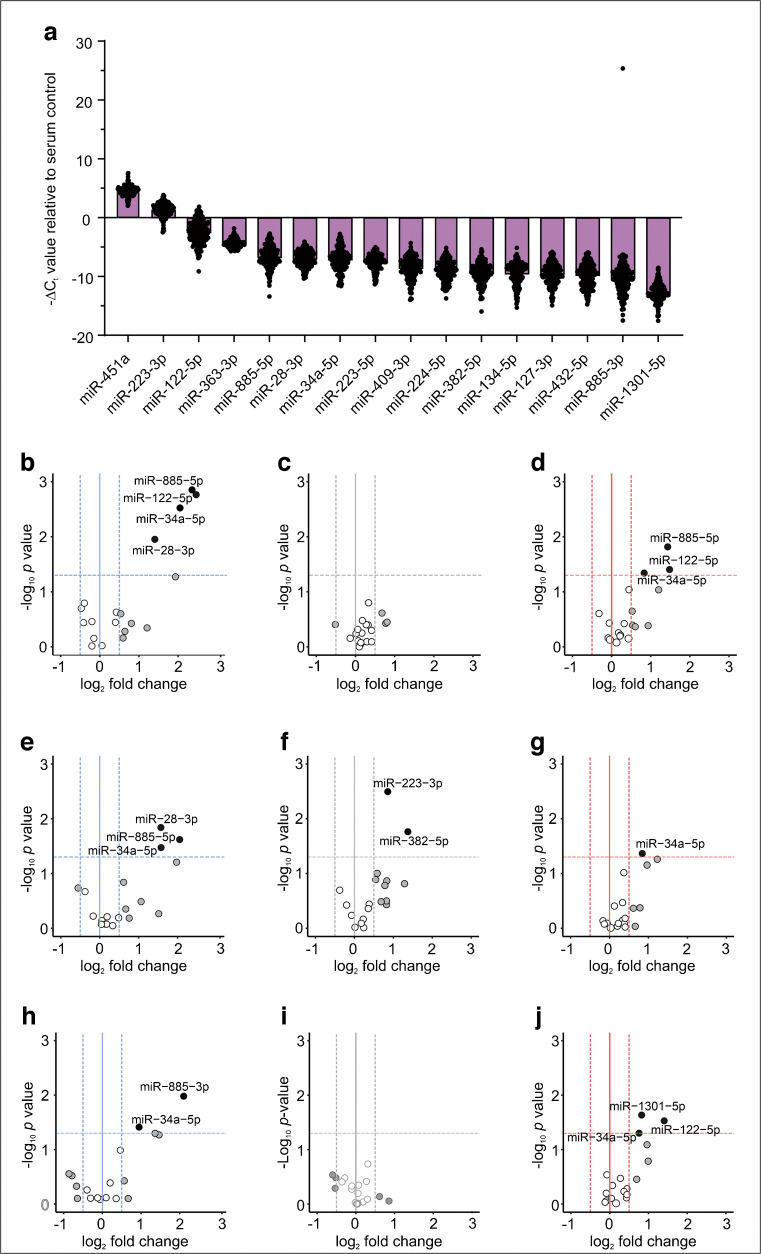
LNA RT-qPCR follow-up of selected microRNAs. (**a**) Overall abundance of circulating microRNAs in the LNA RT-qPCR study regardless of group or timepoint. Expression levels are displayed as −ΔC_t_ values relative to the average of the three endogenous serum controls, miR-16-5p, miR-23a-3p and miR-486-5p. (**b**–**d**) Volcano plots showing pairwise comparisons between NGT and INDET (**b**), IGT (**c**) and CFRD (**d**) at baseline. (**e**–**g**) As in (**b**–**d**), but for the 30 min timepoint. (**h**–**j**) As in (**b**–**d**), but for the 60 min timepoint. Cut-offs at ±1.5-fold change and nominal *p*<0.05 by Student’s *t* test. White dots: no fold change, NS; grey dots: fold change, NS; black dots: fold change and *p*<0.05

In total, 16 microRNAs were selected for analysis across all OGTT timepoints in the overall cohort (93 individuals and 418 samples in total) using a custom-designed LNA primer-based RT-qPCR plate (ESM Table 1). After QC, 210 samples from 53 individuals were included in the final analysis. Regardless of glucose tolerance group or timepoint in the OGTT, miR-451 (used as control for haemolysis), miR-223-3p and miR-122-5p were the three most abundant microRNAs (Fig. 3a). We could further verify elevated serum levels at baseline of miR-34a-5p, miR-122-5p and miR-885-5p, but not miR-885-3p, in the INDET group (Fig. 3b). These three microRNAs were also elevated at baseline in the CFRD group (Fig. 3d).

Next, we assessed the microRNA levels across all timepoints in the OGTT (10, 30, 60 and 180 min) in the INDET, IGT and CFRD groups vs the NGT group (Fig. 3e–j, ESM Fig. 2). In the INDET and CFRD groups, microRNAs with differential expression at baseline (miR-34a-5p, miR-122-5p and miR-885-5p) remained elevated at most timepoints of the OGTT (this is most noticeable in the CFRD group). In addition, miR-1301-5p was upregulated at 60 min in CFRD (Fig. 3j). In the IGT group, miR-223-3p and miR-382-5p exhibited elevated levels at 30 min (Fig. 3f) but not at other timepoints.

In summary, we identified eight microRNAs (miR-28-3p, miR-34a-5p, miR-122-5p, miR-223-3p, miR-223-5p, miR-885-3p, miR-885-5p and miR-1301-5p) with potential as biomarkers and/or possible involvement in CFRD development.

### Three microRNAs were selected for correlation analyses and in vitro follow-up

Based on our data and the published literature, we selected miR-34a-5p, miR-122-5p and miR-223-3p for in-depth assessment (ESM Table 3).

**Fig. 4:**
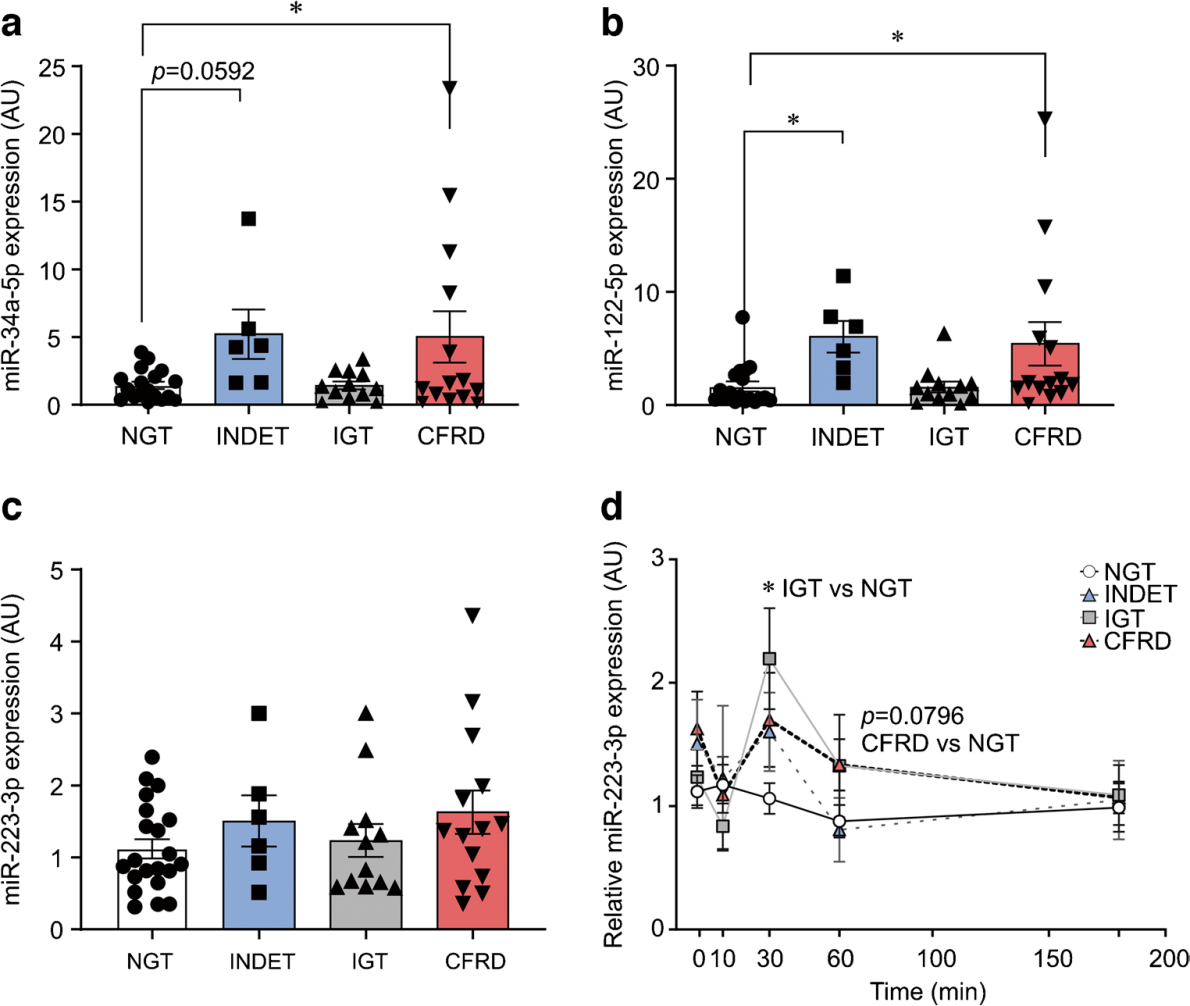
Across-group comparisons and dynamic expression changes for miR-223-3p. (**a**) Bar plot showing the baseline expression for miR-34a-5p. Expression levels are normalised to NGT. NGT, white bar; INDET, blue bar; IGT, grey bar; CFRD, red bar. Error bars show ± SEM. Analysed with one-way ANOVA with Tukey post hoc test, *p<0.05. (**b**, **c**) As in (**a**), but for miR-122-5p and miR-223-3p, respectively. (**d**) Dynamic expression of miR-223-3p throughout the OGTT. Analysed using mixed-effects analysis with uncorrected Fisher, *p<0.05

The serum levels of miR-34a-5p and miR-122-5p were significantly elevated at baseline in the INDET and CFRD groups (Fig. 4a, b). MiR-223-3p levels on the other hand were stable (Fig. 4c), but the dynamic expression of miR-223-3p throughout the OGTT differed markedly between the glucose tolerance groups, most prominently in the IGT group (Fig. 4d).

Since miR-223-3p levels changed during the OGTT, we correlated the levels of this microRNA with glucose and insulin levels during the same OGTT. In the NGT group, miR-223-3p expression was negatively associated with blood glucose at the 30 and 60 min timepoints (ESM Fig. 3a), while circulating insulin and miR-223-3p expression were positively correlated at baseline (ESM Fig. 3b). No other significant associations could be detected.

As both miR-34a-5p and miR-122-5p are secreted from the liver [42, 43], we correlated the baseline expression of these microRNAs with serum liver (aspartate aminotransferase [ASAT], alanine aminotransferase [ALAT]) and bile duct (alkaline phosphatase [ALP], γ-glutamyl aminotransferase [γGT]) damage markers. In CFRD, miR-34a-5p and miR-122-5p levels were positively associated with ASAT, ALAT and γGT (ESM Fig. 4a, b).

The release and/or circulating levels of miR-34a-5p, miR-122-5p and miR-223-3p have been associated with inflammation [44, 45]. Therefore, we examined the correlation of these microRNAs with the inflammatory markers C-reactive protein (CRP) and leukocyte particle concentration (LPK). However, no significant associations were observed (data not shown).

### Bioinformatic microRNA target analysis and overexpression of selected microRNAs

Based on the hypothesis that miR-34a-5p, miR-122-5p and miR-223-3p could be taken up by islet cells to regulate intracellular processes, we developed an in-house bioinformatics pipeline aimed at identifying experimentally validated islet microRNA targets common for all three microRNAs. This generated a list of 18 genes, seven of which are associated with type 2 diabetes (ESM Fig. 5a). Over-representation analysis revealed that the genes primarily are involved in pathways regulating cell division and progression through the cell cycle (ESM Fig. 5a–c).

**Fig. 5:**
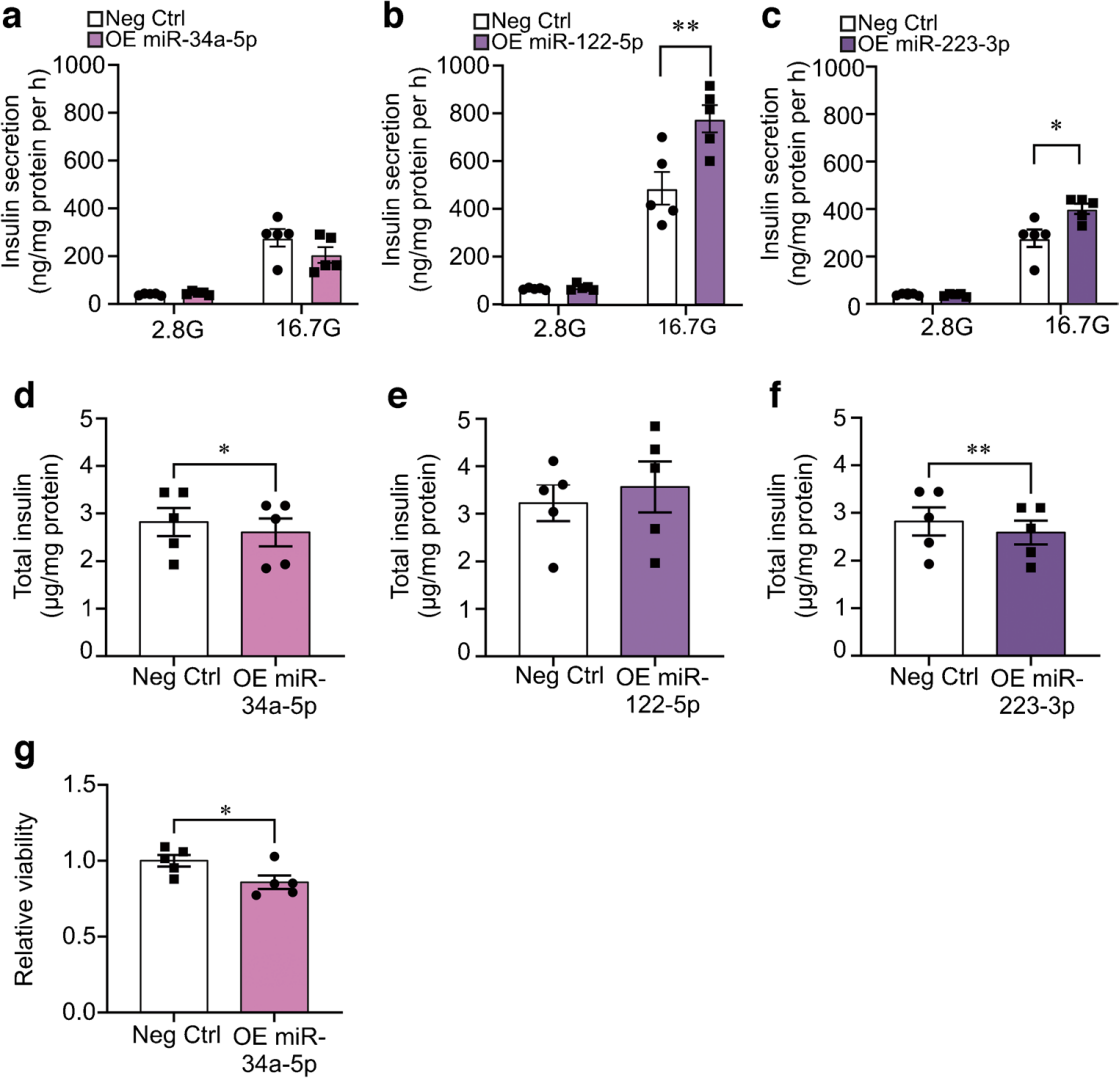
Glucose-stimulated insulin secretion after overexpression of miR-122-5p and miR-223-3p. (**a**–**c**) Glucose-stimulated insulin secretion at low glucose (2.8 mmol/l) and high glucose (16.7 mmol/l) in INS-1 832/13 cells after a 72 h overexpression of miR-34a-5p, (**a**); miR-122-5p, (**b**); and miR-223-3p (**c**), as indicated. White bars shows the negative control microRNA (Neg Ctrl). We ascertained that insulin secretion was significantly elevated in the control at 16.7 mmol/l glucose as compared with 2.8 mmol/l glucose in all experiments (significance not shown). Analysed with two-way ANOVA with Sidak post hoc test, *p<0.05, **p<0.01. (**d**–**f**) As in (**a**–**c**), but for total insulin, analysed with paired Student’s *t* test. (**g**) Relative cell viability (measured using an MTS assay) in INS-1 832/13 cells overexpressing miR-34a-5p. Data are presented as mean ± SEM of *N*=5 independent biological replicates in (**a**–**g**). 2.8G, 2.8 mmol/l glucose; 16.7G, 16.7 mmol/l glucose; OE, overexpression

To mimic a scenario where the circulating microRNAs are taken up by beta cells, we overexpressed miR-34a-5p, miR-122-5p and miR-223-3p in the clonal beta cell line INS-1 832/13 and assessed the effects. Overexpression of miR-122-5p and miR-223-3p improved glucose-stimulated insulin secretion by 60% and 45%, respectively, whereas miR-34a-5p overexpression had no effect (Fig. 5a–c). The increase in insulin secretion was not due to increased insulin content. In fact, insulin content was slightly reduced after overexpression of miR-34a-5p and miR-223-3p (Fig. 5d–f), indicating that the stimulatory effects of the microRNAs are mediated through regulation of proteins in the stimulus–secretion coupling pathway. To further investigate the potential role of miR-34a-5p in beta cell function, we assessed its impact on cell viability. Overexpression of miR-34a-5p in INS-1 832/13 cells led to a significant reduction in viability (Fig. 5g).

## Discussion

In this study we present novel data describing differential expression of circulating microRNAs before and during an OGTT in individuals with CF and varying glucose tolerance. Eight microRNAs (miR-28-3p, miR-34a-5p, miR-122-5p, miR-223-3p, miR-223-5p, miR-885-3p, miR-885-5p and miR-1301-5p) were verified as differentially expressed microRNAs. We specifically identified miR-34a-5p, miR-122-5p and miR-223-3p as highly interesting circulating microRNAs with respect to their ability to function as biomarkers and possible involvement in CFRD development.

MiR-34a-5p and miR-122-5p show elevated serum levels at baseline in the INDET and CFRD groups. Although we cannot determine the source of miR-34a-5p and miR-122-5p, we find a clear correlation with circulating liver damage markers in CFRD, similarly to previous type 2 diabetes studies [14, 46, 47]. MiR-122 is generally considered to be liver specific [42] and it has been reported that the circulating levels of miR-122-5p are elevated in CF liver disease and correlate with liver damage [48]. Indeed, circulating miR-122 is suggested as a biomarker of acute liver failure [42]. In this study both the INDET and the CFRD groups show elevated liver enzymes (Table 2), and high serum miR-122-5p can therefore be indicative of both CFRD development and liver damage. With miR-34a-5p the picture is not as clear as this microRNA is expressed in several major organs, probably reflecting its role in cell cycle regulation. Indeed, miR-34a-5p displays clear expression in healthy human islets in contrast to both miR-122-5p and miR-223-3p [49].

**Table 2:** Clinical characteristics of individuals in the microRNA RT-qPCR study

Characteristic	NGT	INDET	IGT	CFRD
*N* (male/female)	18 (13/5)	6 (4/2)	12 (5/7)	14 (8/6)
Age (years)	27.0 [18.0–42.0]	25.7 [18.0–40.0]	38.4 [24.0–53.0]	32.1 [21.0–45.0]
BMI (kg/m^2^)	23.8 [18.7–39.9]	22.3 [19.5–25.9]	21.2 [17.2–24.3]	22.6 [16.8–27.5]
Insulin (pmol/l)	47.4 [9.9–119.0]	47.4 [37.2–72.5]	31.9 [11.4–62.7]	37.8 [6.0–136.0]
Glucose (mmol/l)	5.3 [5.0–6.8]	5.4 [5.0–5.8]	5.5 [4.2–6.8]	7.7 [4.3–12.8]
CRP (mg/l)	2.9 [1.0–12.0]	2.0 [1.0–7.0]	3.8 [1.0–19.0]	5.4 [1.0–36.0]
LPK (10^6^ cells/l)	6.9 [4.3–10.2]	5.6 [3.9–7.6]	7.0 [3.7–10.1]	7.7 [2.5–14.9]
γGT (U/l)	21.8 [10.0–100.0]	97.2 [10.0–238.0]	37 [7.0–190.0]	105.7 [9.0–767.0]
ALP (U/l)	94.5 [62.0–157.0]	167.0 [55.0–336.0]	112.3 [61.0–335.0]	161.6 [49.0–391.0]
ALAT (U/l)	31.8 [14.0–72.0]	31.5 [15.0–54.0]	28.5 [12.0–57.0]	68.7 [16.0–278.0]
ASAT (U/l)	27.7 [17.0–47.0]	38.2 [16.0–60.0]	30.9 [19.0–75.0]	39.3 [18.0–118.0]

Data are shown as mean [range] at baseline. *N*=50

Circulating microRNAs can be taken up by recipient cells through highly regulated mechanisms and exert regulatory functions in the new host cell [50]. Uptake of miR-122-5p or miR-34a-5p has not been established in islet cells but we show that uptake of miR-122-5p has the potential to significantly increase glucose-stimulated insulin secretion, which would be beneficial in CFRD. Of note, long-standing hyperinsulinaemia exacerbates development of hepatic insulin resistance [51], illustrating the intricate interorgan crosstalk between liver and endocrine pancreas in diabetes development. In contrast, overexpression of miR-34a-5p did not affect insulin secretion but instead reduced beta cell viability.

It has previously been reported that circulating microRNA levels can change within minutes to hours during exercise in humans [52]. The mechanisms behind these rapid changes are not fully understood but could include active secretion of microRNAs, probably as a way of signalling between organs/cells [13], or leaking of microRNAs from damaged cells [53]. In the latter case the microRNAs can function as biomarkers for cell damage. In the context of CF and CFRD development, microRNAs have been highlighted both as possible biomarkers for various stages of the disease and as active players in disease development [54]. Here we show for the first time that circulating microRNA levels (as exemplified by miR-223-3p) can rapidly change during a glucose challenge. In the case of miR-223-3p, the trajectory of the microRNA levels varies with the glucose tolerance state of the individual with CF, but in general miR-223-3p levels increased with glucose ingestion in IGT and CFRD individuals. The implications of this need further investigation, but it is interesting that miR-223-3p targets *CFTR* in bronchial cells [55], where inactivation of this microRNA restores CFTR function [56]. It is therefore possible that increased serum levels of miR-223-3p can have a negative impact on *CFTR* expression throughout the body. The highest expression of miR-223-3p is found in bone tissue and hematopoietic cells [57], but it is unclear whether this is the main source of circulating miR-223-3p in our samples.

It has not been established that circulating miR-223 is taken up by the beta cells, but the literature describes increased islet levels of miR-223 in type 2 diabetes and during glucotoxic stress [58] (the levels of miR-223 are normally very low in healthy islets [49]). In accordance with previous literature [58], we show that overexpression of miR-223-3p improves insulin secretion. In our study, higher levels of serum miR-223-3p correlate with higher circulating fasting insulin levels and lower blood glucose in NGT individuals. However, in individuals with worsened glucose tolerance this association with insulin is lost. It is well established that CF individuals with NGT still have a substantial lifetime risk of developing abnormal glucose metabolism and impaired insulin secretion [59]. Thus, miR-223-3p may serve a compensatory function in individuals with CF by increasing insulin secretion [3, 4, 9–11]. Yet, over time, by targeting *CFTR* in the whole body miR-223-3p could potentially contribute to overall CF progression, which is inherently linked to CFRD development [59].

In conclusion, we identified eight microRNAs with a changed profile before or during an OGTT between the glucose tolerance groups. Specifically, miR-34a-5p and miR-122-5p show potential as biomarkers while miR-223-3p provides proof of concept that the circulating microRNA profile can rapidly change in response to glucose.

All studies have their strengths and limitations. Clearly this study is performed on a limited number of participants and samples were lost due to technical issues. The population of individuals with CFs is inherently small, making the issue of small sample size difficult to address. The strengths of this study include the use of a phenotypically well characterised cohort, an unbiased method, microRNA sequencing, to identify candidate microRNAs, and the novelty of using OGTT samples to investigate the acute serum microRNA changes of a glucose challenge. We believe that this hypothesis-generating study can serve as a solid platform for future investigations into the potential role for circulating microRNAs as diagnostic, monitoring and predictive biomarkers in CFRD.

## Supplementary Information

Below is the link to the electronic supplementary material.

Supplementary file1 (PDF 3423 KB)

Supplementary file2 (XLSX 35 KB)

Supplementary file3 (XLSX 88 KB)

## Data Availability

We cannot readily disclose microRNA sequencing, RT-qPCR or standard laboratory data at an individual level as this is a small participant group with potentially identifiable individuals.

## Abbreviations

ALAT: Alanine aminotransferase
ALP: Alkaline phosphatase
ASAT: Aspartate aminotransferase
CF: Cystic fibrosis
CFRD: Cystic fibrosis-related diabetes
CFTR: Cystic fibrosis transmembrane conductance regulator
CRP: C-reactive protein
FDR: False discovery rate
γGT: γ-Glutamyl aminotransferase
IGT: Impaired glucose tolerance
INDET: Indeterminate glucose tolerance
LNA: Locked nucleic acid
LPK: Leukocyte particle concentration
NGT: Normal glucose tolerance
RT-qPCR: Reverse transcription quantitative PCR
QC: Quality control
UMI: Unique molecular identifier
