# Supplementary material for: Dynamic changes in circulating microRNAs during oral glucose tolerance testing support their potential as diagnostic and monitoring biomarkers in cystic fibrosis-related diabetes
Source: Diabetologia. 2026 Jan 7;69(5):1370–83. doi: 10.1007/s00125-025-06645-7 (PMC13005776; doi:10.1007/s00125-025-06645-7)
Supplement: Supplementary file 1 — Supplementary file1 (PDF 3423 KB) [file 125_2025_6645_MOESM1_ESM.pdf]

Supplementary Table 3: List of microRNAs identified in LNA RT-qPCR follow-up study. Selection for *In vitro* follow-up based on abundance and literature assessment. Related to Figure 3.

**MicroRNAs identified in LNA RT-qPCR**	**MicroRNA-seq result (Baseline/Differential Response)**	**Abundance (High, Medium or Low. Based on Figure 3A)**	**Previous literature. Search performed on 2024-08-01 with term "*miR-xxx AND diabetes*" (PubMed IDs)**	**Comments on selection**
miR-28-3p	Differential Response	Medium	Modest increase in circulating levels of T2D patients (PMID: 20651284).	Excluded.
**miR-34a-5p**	**Baseline**	**Medium**	**Regulates hepatic gluconeogenesis (PMID: 36058294).<br>High levels in circulation of T2D patients (PMID: 27558530, PMID: 20857148)**	**Selected for potential involvement in T2D**
**miR-122-5p**	**Baseline**	**High**	**Liver enriched miRNA, associated with liver damage (PMID: 30087537).<br>Associated with CF liver disease (PMID: 25625579)**	**Selected for high levels. Association with CF liver disease.**
**miR-223-3p**	**Differential Response**	**High**	**Upregulated in islet from obese and T2D patients and maintains functional β-cell mass PMID: 31118273.<br>High levels in circulation predict progression to T2D (PMID: 31721085)**	**Selected for high levels.<br>Implications for β-cell function.<br>Can also target CFTR (PMID: 32059764, PMID: 23436935).**
miR-223-5p	Differential Response	Medium	No relevant studies found.	Excluded.
miR-885-3p	Baseline	Low	Downregulated in mononuclear cells from peripheral blood in T1D patients PMID: 31763742).	Excluded due to low levels
miR-885-5p	Baseline	Medium	Upregulated in amniotic fluid in GDM pregnancies (PMID: 31852997).	Excluded
miR-1301-5p	Differential Response	Low	No relevant studies found.	Excluded due to low levels

Extra Supplementary Material (ESM) Westholm *et al*

ESM Figures

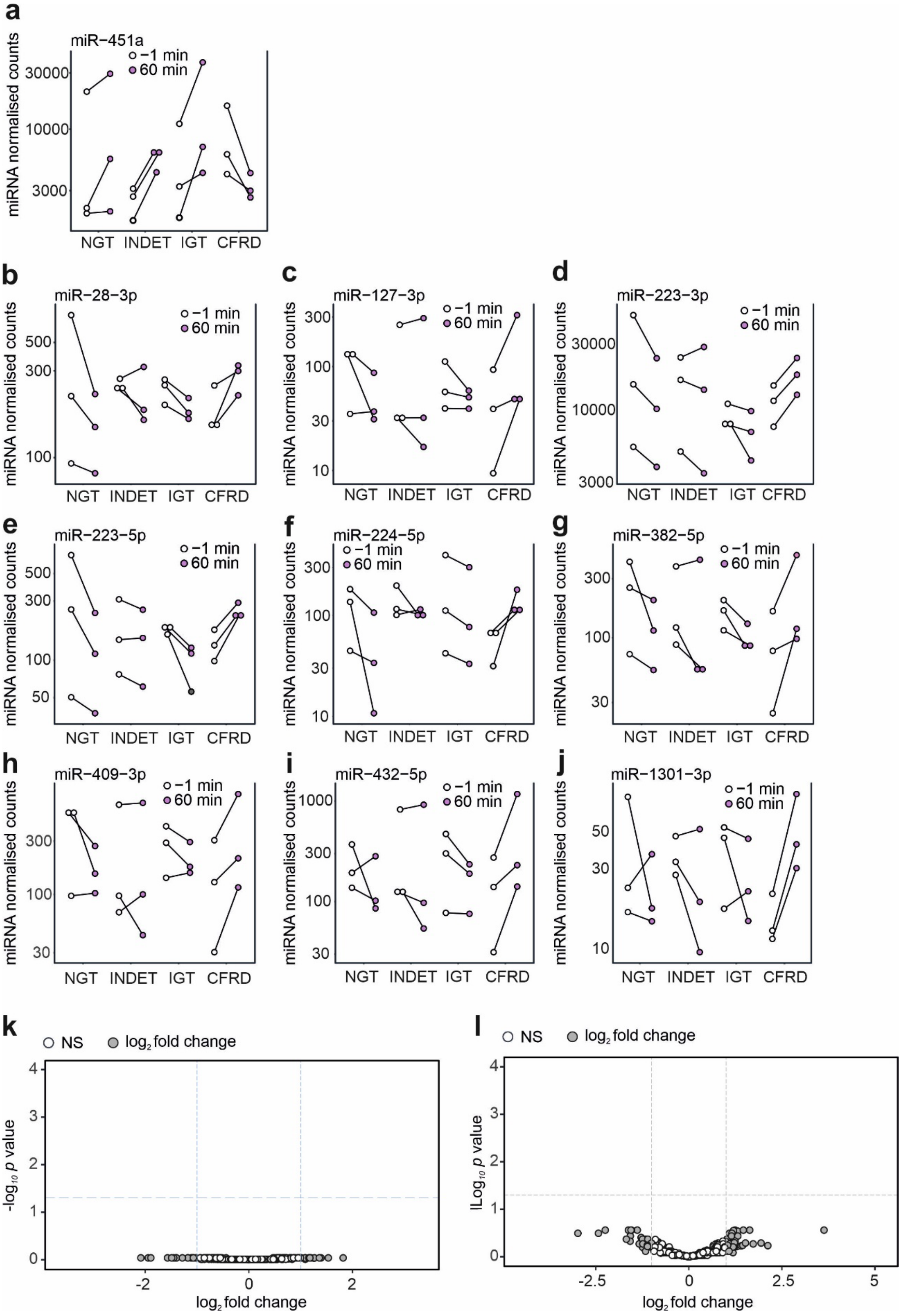

ESM Fig. 1: Additional characteristics of microRNAs identified in differential response analysis. Related to Figure 2.

(a) Dot plots showing dynamic expression changes for miR-451a. This microRNA has a negative response in differential response analysis (Fig. 2b). **(b-j):** Dot plots showing dynamic expression changes for miR-28-3p, miR-127-3p, miR-134-5p, miR-223-3p, miR-223-5p, miR-224-5p, miR-382-5p, miR-409-3p, miR-432-5p and miR-1301-3p. These microRNAs have a positive response in differential response analysis (Fig, 2b). For (a-j): Connected dots show individual patients within the four groups. white dots show the baseline (-1 min) and purple dots show the 60- min time point. **(k-l):** Volcano plots showing microRNAs differentially responding to glucose intake between NGT *vs* INDET **(k)** and NGT *vs* IGT **(l).** Cut-offs at ±2 fold change and FDR-adjusted $P<0.05$. White dots: no fold change, NS, grey dots: fold change, NS.

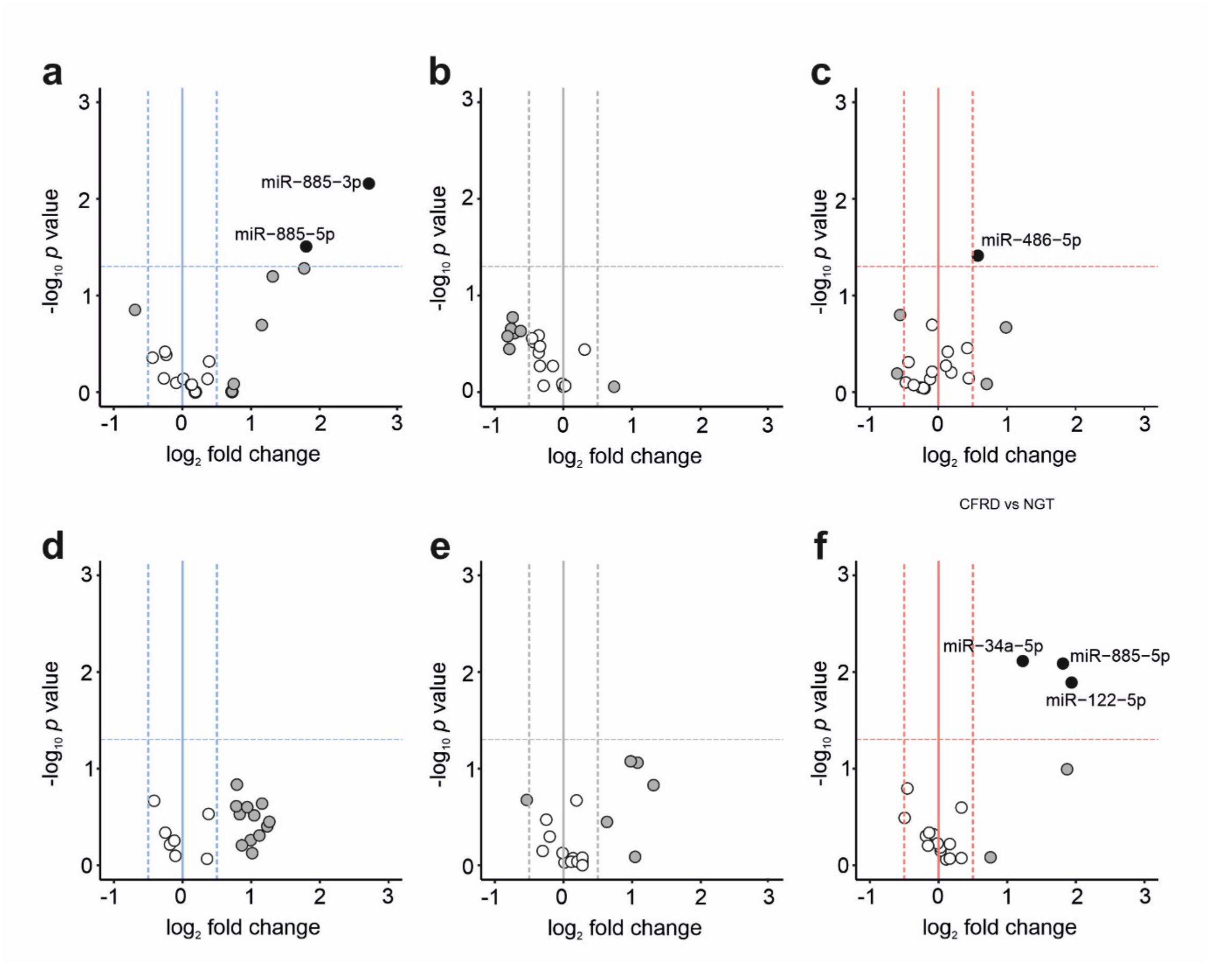

ESM Fig. 2: Volcano plots for time points 10-min and 180-min in differential response analysis in LNA RT-qPCR follow-up study. Related to Figure 3.

(a-c): Volcano plots showing pairwise comparisons at 10-min between the four groups NGT and **(a)** INDET **(b)** IGT and **(c)** CFRD. Cut-offs at ±1.5 fold change and $P<0.05$ by Student's T-test. White dots: no fold change, NS; grey dots: fold change, NS; black dots: fold change and $p<0.05$. **(d-f)**: as in (a-c), but for time-point 180-min.

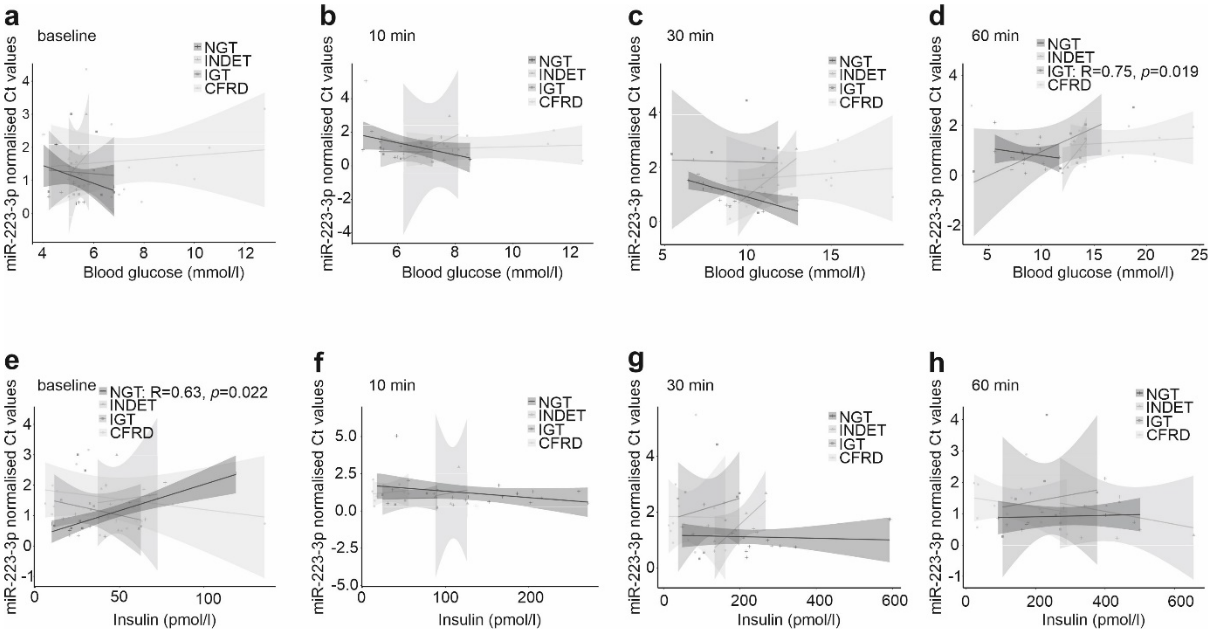

ESM Fig. 3: Spearman correlations between miR-223-3p, blood glucose and insulin. Related to Figure 4.

(a-d) Spearman correlations between miR-223-3p and blood glucose in the four categories NGT, INDET, IGT and CFRD at four time-points: **(a)** baseline (-1 min)**,** (b) 10-min, **(c)** 30-min and **(d)** 60-min. Shaded areas represent the Spearman correlation with its 95%CI. Darkest shade of grey is NGT, lightest shade of grey is CFRD. Significant correlations are noted. **(e-f):** As in (a-d), but correlations are for miR-223-3p and insulin levels.

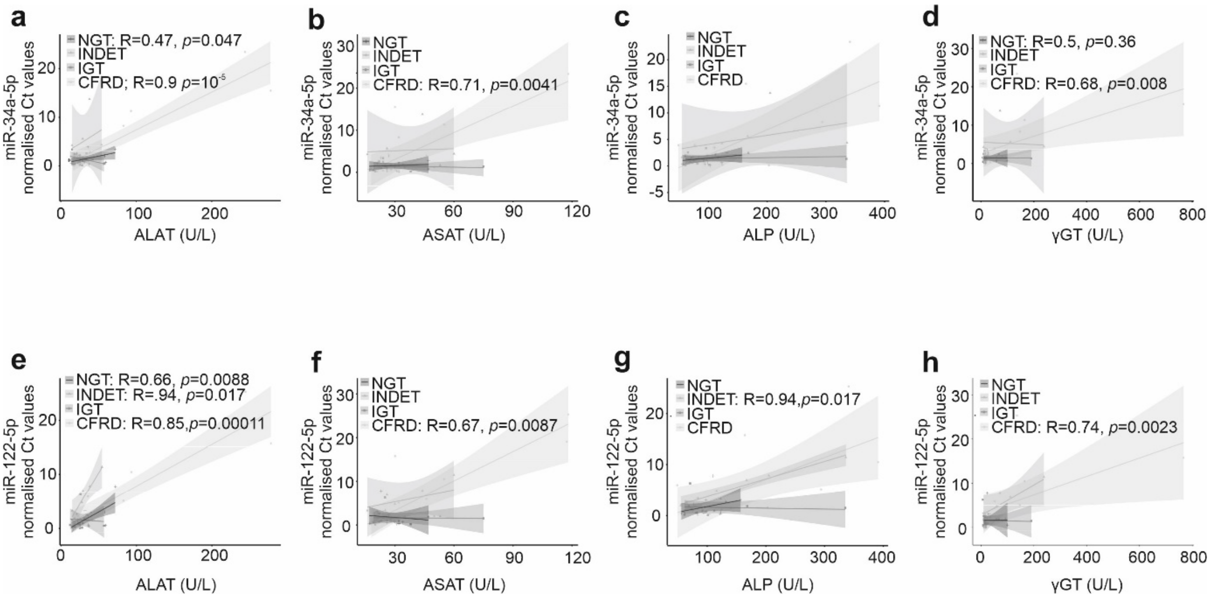

ESM Fig. 4: Spearman correlations between miR-34a-5p and miR-122-5p and hepatobiliary markers. Related to Figure 4.

(a-d): Spearman correlations between miR-34a-5p and hepatobiliary markers in the four categories NGT, INDET, IGT and CFRD: **(a)** ALAT, **(b)** ASAT, **(c)** ALP and **(d)** γGT. Shaded areas represent the Spearman correlation with its 95%CI. Darkest shade of grey is NGT, lightest shade of grey is CFRD. Significant correlations are noted. **(e-h)**: As in (a-d), but correlations are for miR-122-5p and hepatobiliary markers.

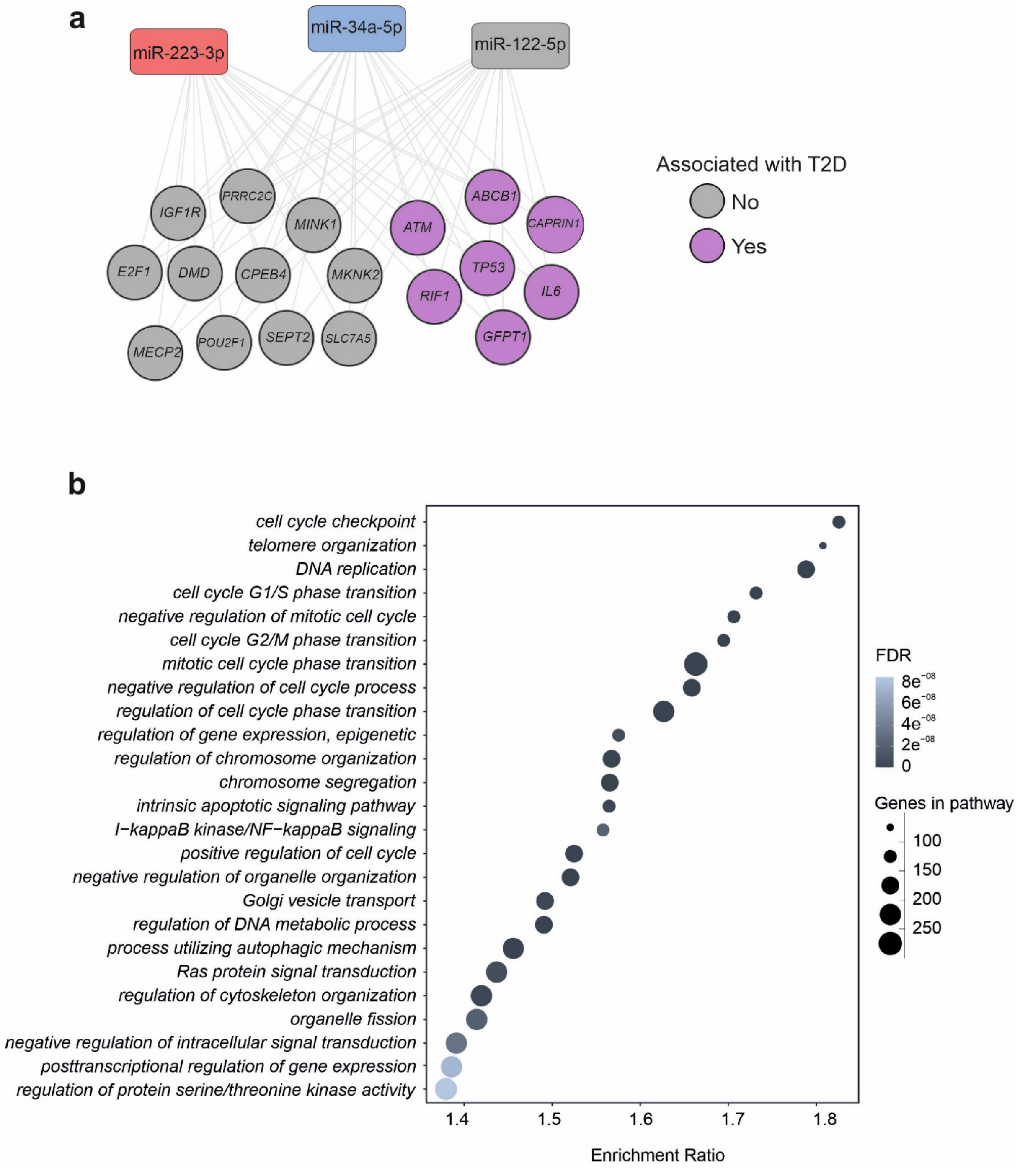

ESM Fig. 5: Bioinformatic analysis of gene targets for miR-34a-5p, miR-122-5p and miR-223-3p. Related to Figure 4.

(a): Common gene targets for miR-34a-5p, miR-122-5p and miR-223-3p. Targets not associated with type 2 diabetes (T2D) in light grey circles and targets associated with T2D are in purple circles. **B:** Pathway overrepresentation analysis of combined targets for miR-34a-5p, miR-122-5p and miR-223-3p. Enrichment ratio on the x-axis, size of the circle represents number of genes in the pathway, and shade of blue represents FDR-adjusted P-value for enrichment significance.
